# A patient presented with Late onset adrenoleukodystrophy as Dementia

**DOI:** 10.1002/alz70857_103218

**Published:** 2025-12-25

**Authors:** Murat Gultekin

**Affiliations:** ^1^ Erciyes University School Of Medicine, Department of Neurology, Kayseri, Turkey

## Abstract

**Background:**

X‐linked Adrenoleukodystrophy (X‐ALD) is the most common leukodystrophy and peroxisomal disease with an incidence ranging from 1:15,000–50,000 males [2,3]. X‐ALD results from a variety of mutations of the ABCD1 gene (ATP‐binding cassette, D1 subtype) in the Xq28 chromosome. For late‐onset rarely presentation in adults such as the previously determined that do not shown typical childhood symptoms, later in life present cognitive problems like dementia and behavioral changes.

**Method:**

In the genetic analysis, ABCD1 gene exon 1 c,761C>T and p.T254M homozygous were detected.

**Result:**

The 47‐year‐old male patient had a cognitive performance decline that started 12 years ago. Therefore, he began to experience occupational loss. In the following years, gait disorder and vision problems emerged. In the patient's neurological examination, spasticity was detected in the lower extremities. The DTR response was extensor. Visual acuity was significantly impaired and he could count fingers from 1 meter. In the following years, the patient developed mutism and became bedridden as his neurological condition progressed. No significant disorder was detected in the patient's endocrine tests. In the brain MRI, widespread white matter signal change and significant global cortical atrophy were detected.

**Conclusion:**

The X‐ALD is rarely presents in adults also with a progressive white matter affection and it could be presented differ clinical manifestations such dementia.

